# Effect of *Saccharina japonica* Intake on Blood Pressure and Gut Microbiota Composition in Spontaneously Hypertensive Rats

**DOI:** 10.3390/microorganisms12030556

**Published:** 2024-03-11

**Authors:** Ayaka Harui, Saki Maruyama, Yukiko Segawa, Nobutaka Kurihara

**Affiliations:** 1 Hygiene and Preventive Medicine, Graduate School of Home Economics, Kobe Women’s University, 2-1 Higashisuma-Aoyama, Suma, Kobe 654-8585, Japan; haruiayaka@gmail.com (A.H.); s-maruyama@suma.kobe-wu.ac.jp (S.M.); segawa@osaka-seikei.ac.jp (Y.S.); 2 Faculty of Cookery and Confectionery, Osaka Seikei College, 10-62 Aikawa, Higashiyodogawa, Osaka 533-0007, Japan

**Keywords:** *Saccharina japonica*, antihypertensive effect, gut microbiota

## Abstract

It was reported that the consumption of *Saccharina japonica* (SJ) lowers blood pressure (BP) in hypertensive rats. Hypertension is related to gut microbiota, and hypertensive patients develop dysbiosis. It was reported that the intake of dietary fiber and polysaccharides contained in SJ changes gut microbiota and increases short-chain fatty acids (SCFAs). The present study examined the effect of BP lowering by SJ in spontaneously hypertensive rats (SHRs) and observed changes in gut microbiota composition and SCFAs concentration. Male SHRs and Wistar Kyoto rats (WKYs) were fed a diet containing 5% SJ or a control diet for six weeks. We measured systolic BP (SBP) weekly, as well as mean arterial BP (MAP), the 16S rRNA gene, and SCFAs in the cecal contents at the end of the period. As a result, the intake of SJ significantly decreased SBP and MAP in SHRs. As well, it significantly changed the microbial diversity by altering the gut microbiota composition. Particularly, it increased the abundance of *Bacteroides acidifaciens*, which may be associated with the antihypertensive effect of SJ. Thus, SJ intake suppressed the increase in BP and altered the gut microbiota composition, although it did not significantly change the SCFAs concentration in the cecal contents.

## 1. Introduction

Hypertension causes stroke, coronary heart disease, and renal failure [1,2,3]. The factors contributing to blood pressure (BP) elevation include the activation of sympathetic nerves, the renin-angiotensin-aldosterone system (RAAS), and endothelial dysfunction. Previous studies on the BP-lowering effects of ingesting foods and their ingredients have reported angiotensin converting enzyme (ACE) inhibition due to ACE inhibitory peptides, as well as antioxidant effects [4,5]. Previous studies have observed that the intake of *Saccharina japonica* (SJ) suppressed the increase in BP in spontaneously hypertensive rats (SHRs) and 2-kidney, 1-clip renovascular hypertensive (2K1C) rats [6,7]. However, the mechanism behind the antihypertensive effect of SJ intake remains unclear, although it has been suggested to involve ACE inhibitory activity in vitro [8].

SJ is a type of brown algae commonly known as kombu. The dietary fibers contained in SJ include alginate, fucoidan, and laminaran [9]. Notably, it contains the highest amount of alginate. A previous report showed that the administration of potassium alginate lowered BP in SHRs and induced changes in gut microbiota [10]. As well, we previously observed that the intake of sodium alginate lowered BP in 2K1C rats and restored intestinal barrier function [11]. Furthermore, it was reported that alginate, fucoidan, and laminaran, when administered separately, altered the gut microbiota in normotensive rats and modulated the concentration of metabolites in the intestines [10,12,13,14].

The short-chain fatty acids (SCFAs), including acetate, butyrate, and propionate, are produced by bacteria through fermentation in the colon, which have been shown to reduce BP in normotensive and hypertensive models when administered orally, intracolonically, intravenously, or intrarenally [15,16,17,18,19,20,21]. The mechanism is suggested to involve vasodilation and inhibition of sympathetic nerves via SCFA receptors [18,22,23,24]. It has been reported that the activation of G protein-coupled receptors (GPRs) 41 and GPR 43 leads to a decrease in BP [18,22], while the activation of Olfactory receptor (Olfr) 78 mediates renin secretion and increases BP [25]. 

Recent studies have focused on the relationship between gut microbiota and diseases and revealed the associations of hypertension, arteriosclerosis, and coronary artery disease with gut microbiota [26,27,28,29]. Other studies demonstrated a significant increase in BP when feces and cecal contents from hypertensive model rats were transplanted into normotensive rats [23,30,31]. Moreover, a reduction in the number and diversity of SCFAs-producing bacteria was observed in the gut microbiota of SHR compared to the control normotensive model, Wistar Kyoto rats (WKYs) [29,32,33]. It has also been reported that reducing intestinal bacteria through antibiotic administration lowered BP in angiotensin II-induced hypertensive models, salt-sensitive hypertensive models, and SHRs [29,34,35]. 

Additionally, probiotics, known for their beneficial effects on host health, have been reported to decrease in the intestinal flora of hypertensive model rats compared with normotensive controls. It was reported that Lactobacillus strains (*Lactobacillus fermentum* or *L. coryniformis* plus *L. gasseri*) lowered systolic BP (SBP) and improved endothelial function when administered to SHRs [36]. It was also reported that *Clostridium butyricum* lowered SBP and prevented the SCFAs reduction and inflammation in the cecum and plasma in SHRs [37]. 

Therefore, we hypothesized that one of the mechanisms by which the intake of SJ suppresses the increase in BP in SHRs is related to the modulation of the abundance of SCFAs-producing bacteria and the production of SCFAs by dietary fibers of SJ. In this study, to test the hypothesis, we observed changes in the gut microbiota and SCFAs production in the colon after confirming the suppression of BP elevation in SHRs by SJ intake.

## 2. Materials and Methods

### 2.1. Animals and Treatment

Four-week-old male SHRs and WKYs were purchased from Japan SLC (Shizuoka, Japan) and were kept in a temperature and moisture-controlled room (21 ± 1 °C, 50 ± 10% in a 12 h light/dark cycle). The rats had free access to a standard chow (CE-2; CLEA, Tokyo, Japan) and tap water during a preliminary breeding period of 2 weeks. The protocol was approved by the Animal Experiment Committee of Kobe Women’s University (A257 on 30 April 2021; A257–1 on 29 October 2021).

After the preliminary breeding period, each model was divided randomly into two groups and administered either a control diet (CTL diet) or a 5% (5 of 100, *w*/*w*) SJ-containing diet (SJ diet), with free access to drinking water, for 6 weeks. Thus, the rats were divided into four groups: (1) WKYs fed a CTL diet (WKY-CTL), (2) WKYs fed an SJ diet (WKY-SJ), (3) SHRs fed a CTL diet (SHR-CTL), and (4) SHRs fed an SJ diet (SHR-SJ). The amounts of intake, body weight, and SBP of each group were observed during the feeding period. SBP was measured weekly using the tail-cuff method without anesthesia for 6 weeks. At the end of the breeding period, mean arterial BP (MAP) was measured under anesthesia. After that, the rats were euthanized, and the contents of the cecum were collected and stored at −80 °C.

### 2.2. The Experimental Diets

Teklad Global Diet 2014 (Envigo, Inc., Indianapolis, IN, USA) was used as a standard diet, and by mixing this diet with SJ at a ratio of 95:5, an SJ diet was prepared. SJ was harvested in Hokkaido, Japan. The salt and dietary fiber contents in 100 g of SJ were 0.57 g and 5.5 g, respectively. For a CTL diet, the standard diet was supplemented with NaCl (FUJIFILM-WAKO, Osaka, Japan) and cellulose powder (CLEA Japan, Inc., Tokyo, Japan), to match the salt and dietary fiber contents of the SJ diet.

### 2.3. Blood Pressure Measurements

SBP was evaluated as previously described [38,39]. Briefly, it was measured using a tail-cuff method utilizing the MK-1030 NIBP monitor (Muromachi Kikai Co., Ltd., Tokyo, Japan) in conscious animals every week. Before the measurements, the rats were restrained and placed in a chamber at 38 °C for 10 min to make the detection of arterial pulsation in the tail and to stabilize their SBP. SBP was measured consecutively 10 times, with the average value used for evaluation.

MAP measurement was performed by the method described previously [38,39]. The rats were anesthetized with a mixture of 0.15 mg/kg medetomidine hydrochloride, 2 mg/kg midazolam, and 2.5 mg/kg butorphanol injected intraperitoneally. The left femoral artery was catheterized with polyethylene tubing (PE-10; Becton Dickinson, Sparks, MD, USA) attached to a pressure transducer and recorder. The MAP was continuously monitored using a PowerLab System/ 8sp with a signal amplifier (AD Instruments, Belle Vista, Australia) connected to a BP transducer (AR611G; Nihon Kohden Corp., Tokyo, Japan). The data were collected during the last 3 min of a 10 min stabilized period.

### 2.4. 16S rRNA Gene Extraction and Sequencing

The cecal contents of SHRs were collected after euthanasia and analyzed for microbiota through 16S ribosomal RNA amplicon sequencing, utilizing the V3-V4 hypervariable regions. The composition of gut microbiota was analyzed using QIIME2 (ver. 2021.11) [40]. Bacterial DNA was extracted using the QIAamp DNAPowerFecal DNA kit (Qiagen Inc, Venlo, The Netherlands), following the manufacturer’s protocol. To analyze the V3-V4 region, the initial PCR used V3V4f_MIX (ACACTCTTTCCCTACACGACGCTCTTCCGATCTNNNNNCCTACGGGNGGCWGCAG) and V3V4r_MIX (GTGACTGGAGTTCAGACGTGTGCTCTTCCGATCTNNNNNGACTACHVGGGTATCTAATCC) primers. The Premix Ex Taq^TM^ Hot Start Version (Takara Bio Inc., Kusatsu, Shiga, Japan) was employed for PCR, conducted under the following conditions: an initial denaturation at 94 °C for 2 min, followed by 25 cycles of 94 °C for 30 s, 55 °C for 30 s, and 72 °C for 30 s, with a final extension step at 72 °C for 5 min. The second amplification was performed using index primers. Sequencing was conducted using the Illumina MiSeq sequencing system (Illumina Inc., San Diego, CA, USA) and the MiSeq Reagent Kit v3 (2 × 300 bp) chemistry (Illumina Inc.), which was conducted by Bioengineering Lab. Co., Ltd. (Sagamihara, Japan). QIIME2 version 2021.11, with default parameters, was used for sequence denoising using the DADA2 method for chimera checking. The Operational Taxonomic Units (OTUs) table was clustered with a 97% similarity cutoff based on the open-reference approach using Greengene (ver. 13_8). 

### 2.5. Measurements of SCFAs

The SCFAs in the cecal contents were determined using the method described by Takagi et al. with minor modifications [41]. For measuring SCFAs, 0.1 g of the rats cecal contents was placed in a 2.0 mL tube with zirconia beads and suspended in Milli-Q water. The samples were heated at 85 °C for 15 min, vortexed at 5 m/s for 45 s using FastPrep 24 5G (MP Biomedicals, Santa Ana, CA, USA), and then centrifuged at 15,350× *g* for 10 min. The resulting supernatant was filtered by a membrane with a pore size of 0.20 µm.

A high-performance liquid chromatography (Prominence, Shimadzu, Kyoto, Japan) was employed for the measurement of organic acids in cecal contents, including acetate, propionate, butyrate, succinate, and lactate. The detection was performed through a post-column reaction with a detector (CDD-10Avp, Shimadzu), utilizing tandemly arranged threecolumns (Shim-pack Fast-OA, 100 mm × 7.8 mm ID, Shimadzu), and a guard column (Shim-pack Fast-OA, 10 mm × 4.0 mm ID, Shimadzu). The system used a mobile phase (5 mM p-toluenesulfonic acid) and a reaction solution (5 mM p-toluenesulfonic acid, 100 µM EDTA, and 20 mM Bis-Tris). The flow rate and oven temperature were set at 0.8 mL/min and 50 °C, respectively. The measurement was conducted by TechnoSuruga Labo., Ltd. (Shizuoka, Japan).

### 2.6. Statistical Analysis

SBP, MAP, and SCFAs concentration were expressed as mean ± standard error (SE). Analysis of variance (ANOVA) tests were conducted to compare BP and SCFAs concentration among the groups. The Mann–Whitney U test was employed to compare the alpha diversity index and the relative abundance level of microbes between each group. Permutational multivariate analysis of variance (PERMANOVA) was performed to detect significant differences in beta diversity. The Wilcoxon rank-sum test was also utilized in linear discriminant analysis effect size (LEfSe) to identify bacterial biomarkers that significantly differed in abundance between the groups using Galaxy module [42]. These statistical analyses and bioinformatics analyses of the microbial data were conducted using SPSS statistics version 23.0 (IBM, Chicago, IL, USA) and R 4.3.0. A *p*-value of 5% or lower was considered statistically significant.

## 3. Results

### 3.1. Effect of Saccharina japonica Intake on SBP and MAP in SHRs

**Figure 1:**
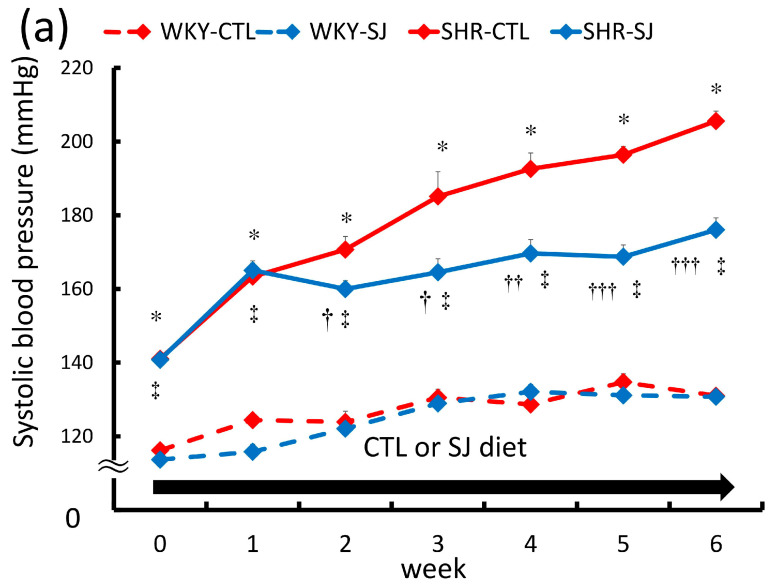

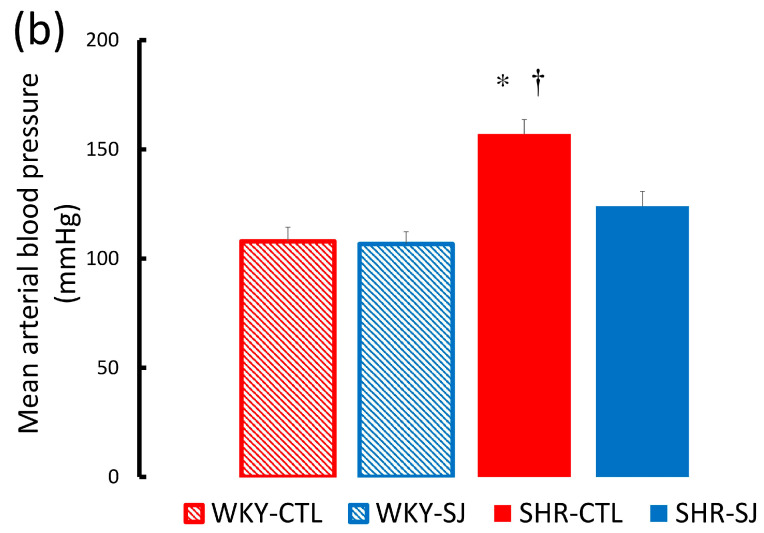
Systolic blood pressure (**a**) and mean arterial blood pressure (**b**) in WKYs or SHRs fed CTL or SJ for 6 weeks. Values are mean ± SE, *n* = 4–5. (**a**) Three-way ANOVA: *p* < 0.001 for time, animal (WKYs vs. SHRs), and diet and each interaction except time × diet (*p* < 0.01). * *p* < 0.001 vs. WKY-CTL, ^†^ *p* < 0.05, ^††^ *p* < 0.01, and ^†††^ *p* < 0.001 vs. SHR-CTL, ^‡^ *p* < 0.001 vs. WKY-SJ. (**b**) Two-way ANOVA: *p* < 0.0001 animal, *p* < 0.05 diet and interaction animal × diet. * *p* < 0.01 vs. WKY-CTL, ^†^ *p* < 0.05 vs. SHR-SJ. The following abbreviations were used: WKY, Wistar Kyoto rats; SHR, spontaneously hypertensive rats; CTL, a control diet; SJ, a diet with *Saccharina japonica*; SE, standard error; ANOVA, analysis of variance.

The SBP of SHR-CTL was significantly higher than that of WKY-CTL (Figure 1a). The SBP of SHR-SJ was significantly lower than that of SHR-CTL. There was no significant difference in SBP between WKY-SJ and WKY-CTL. Similarly, the MAP of SHR-CTL was significantly higher than that of WKY-CTL (Figure 1b). SHR-SJ showed significantly lower MAP than SHR-CTL. There was no significant difference in MAP between WKY-CTL and WKY-SJ. As observed in a previous study [6], chronic SJ intake significantly suppressed the elevation of BP in SHRs in the present study.

### 3.2. Effects of Saccharina japonica Intake on the Microbial Taxonomic Composition in SHRs

**Figure 2:**
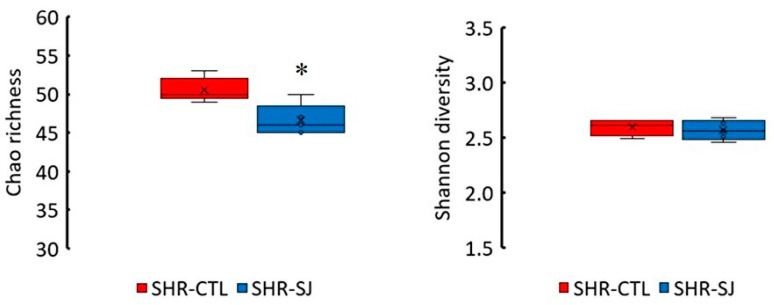
Box plots of the alpha diversity (Chao1, Shannon) in SHRs fed a CTL or SJ diet. Cross reports mean, horizontal black line represents median, and upper and lower boundaries of box represent 75 th and 25 th percentile, respectively. Vertical line represents 95% confidence interval for the median. *n* = 5 per group. * *p* < 0.05 vs. SHR -CTL by Mann–Whitney U test. See legend of Figure 1 for abbreviations.

**Figure 3:**
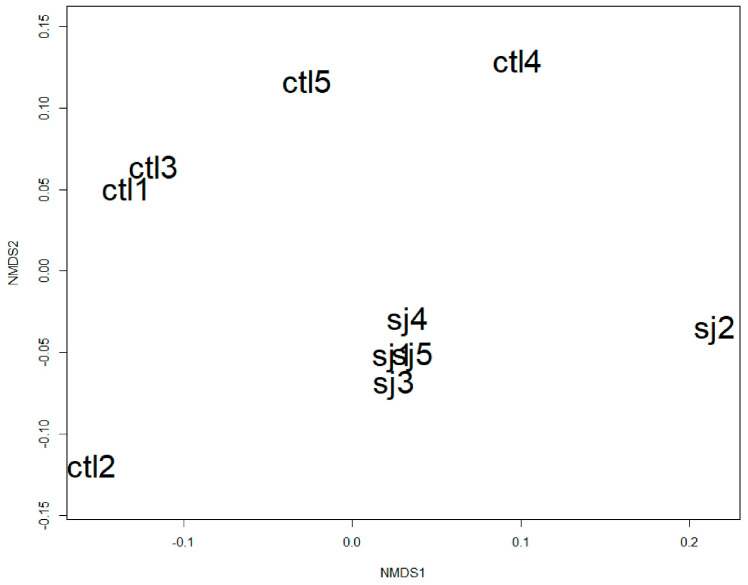
Beta diversity of gut microbes analyzed by NMDS based on Bray-Curtis dissimilarity in SHRs fed CTL (ctl 1–5) or SJ (sj 1–5). *p* < 0.05 by permutational multivariate analysis of variance, *n* = 5 per group. The following abbreviations were used: NMDS: Non-metric multi-dimensional scaling; SHR, spontaneously hyperten-sive rats; CTL, a control diet; SJ, a diet with *Saccharina japonica*.

Alpha diversity analysis revealed that bacterial richness (Chao1) in SHR-SJ was lower than that in SHR-CTL in terms of amplicon sequence variants abundance. However, there were no significant differences in diversity according to the Shannon index (Figure 2). Non-metric multi-dimensional scaling (NMDS) based on Bray–Curtis distance indicated significant distinctions in the microbiota communities within SHR-CTL and SHR-SJ (Figure 3).

**Figure 4:**
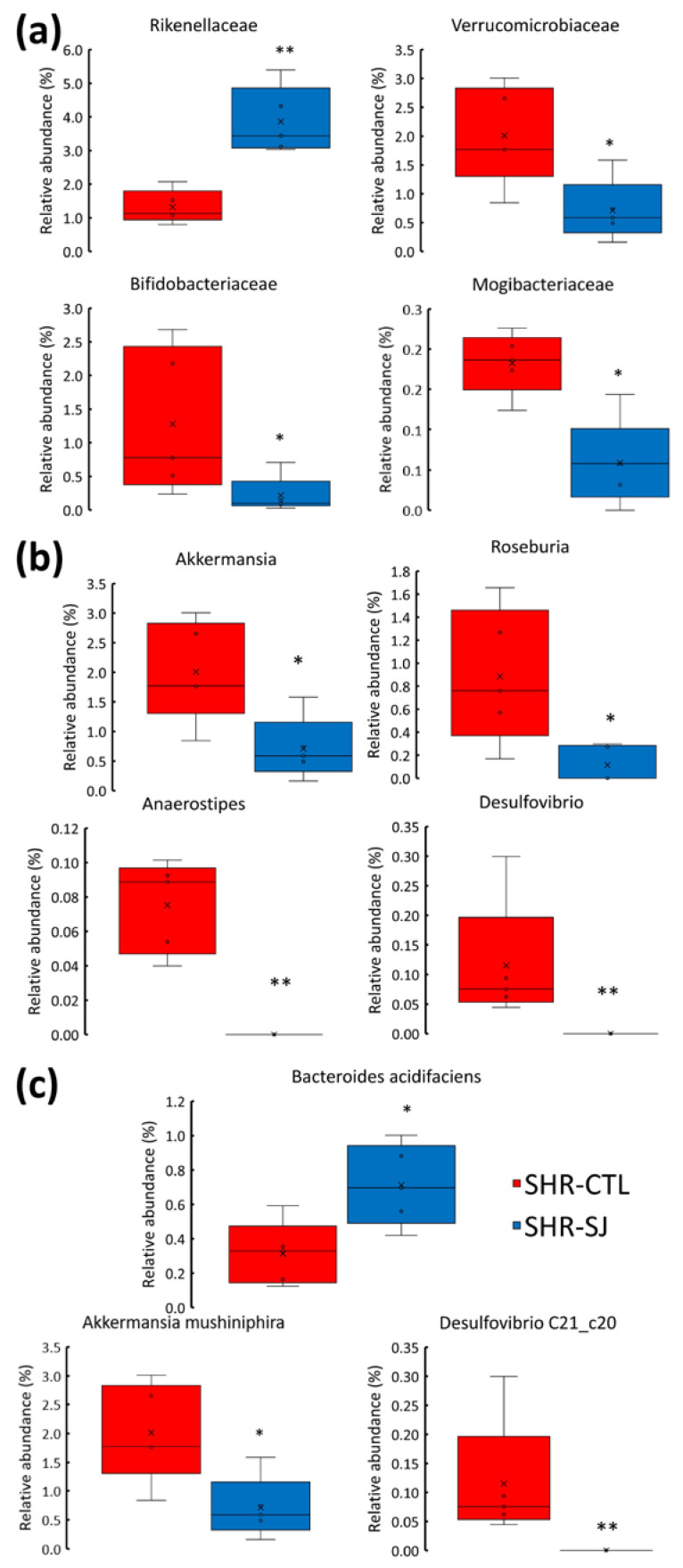
Effects of SJ on gut microbiota composition at the differential abundance of (**a**) family, (**b**) genus, (**c**) species in SHRs fed CTL or SJ at the end of feeding. Cross reports mean, horizontal black line represents median, and upper and lower boundaries of box represent 75 th and 25 th percentile, respectively. Vertical line represents 95% confidence interval for the median. *n* = 5. * *p* < 0.05, ** *p* < 0.01 vs. SHR-CTL by Mann–Whitney U test. See legend of Figure 1 for abbreviations.

**Figure 5:**
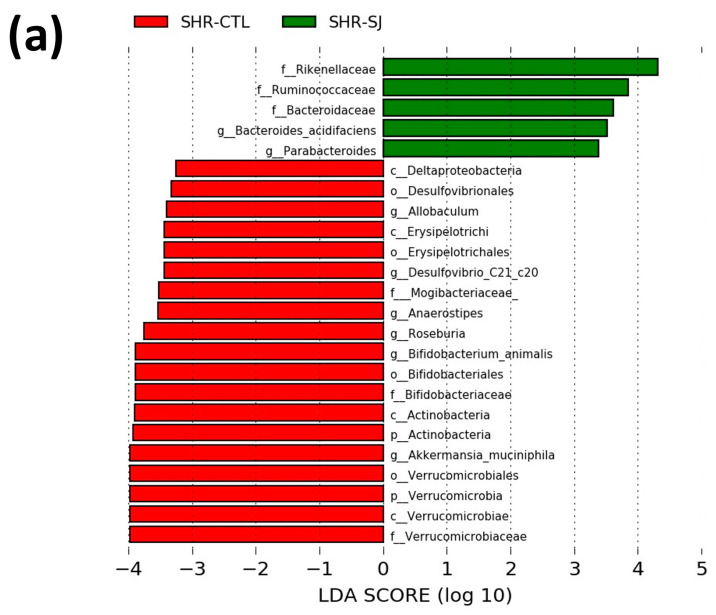

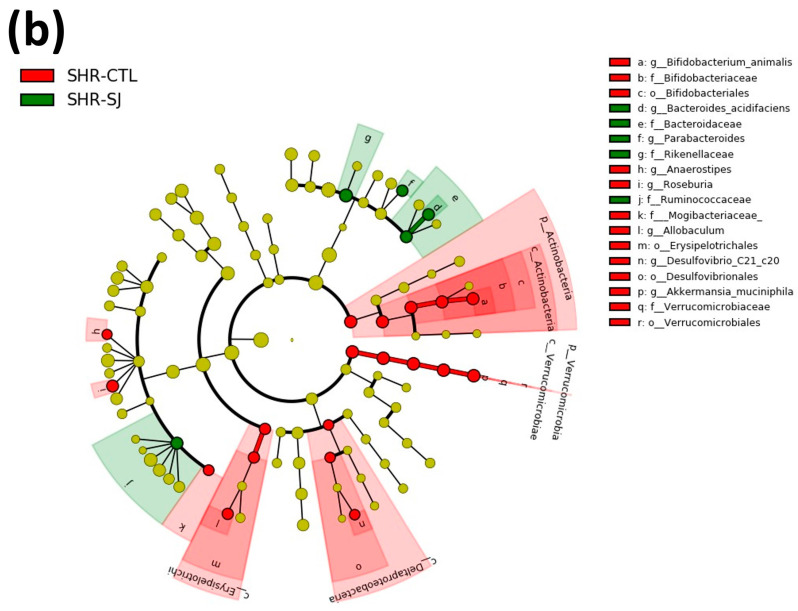
The histogram of the linear discriminant analysis value distribution gut microbiota (**a**) and cladograms (**b**) in SHRs fed a CTL or SJ diet. *p* < 0.05 by the Wilcoxon rank-sum test, *n* = 5 per group.

The relative abundances of *Firmicutes*, *Bacteroidetes*, and *Proteobacteria* were not significantly changed with the intake of an SJ diet in SHRs. In contrast, *Verrucomicrobia* and *Actinobacteria* were significantly decreased in SHR-SJ compared with SHR-CTL (*p* < 0.05, Table 1). At the family level, the relative abundance of *Rikenellaceae* significantly increased in SHR-SJ compared with SHR-CTL. Conversely, the relative abundances of *Verrucomicrobiaceae*, *Mogibacteriaceae*, and *Bifidobacteriaceae* significantly decreased in SHR-SJ compared with SHR-CTL (Figure 4a). At the genus level, the relative abundances of *Roseburia*, *Anaerostipes*, *Akkermansia*, and *Desulfovibrio* were significantly decreased in SHR-SJ compared with SHR-CTL (Figure 4b). At the species level, *Bacteroides acidifaciens* significantly increased in SHR-SJ compared with SHR-CTL. In contrast, the relative abundances of *Akkermansia mushiniphira* and *Desulfovibrio* C21_c20 were significantly decreased in SHR-SJ compared with SHR-CTL (Figure 4c). Furthermore, LEfSe analysis revealed that the relative abundances of 5 bacterial taxa were significantly increased, and 19 bacterial taxa were significantly decreased in SHR-SJ compared with SHR-CTL (Figure 5). These data indicate that SJ consumption may alter the composition of gut microbiota in SHR.

### 3.3. Effects of Saccharina japonica on the SCFAs Concentration in SHRs 

Contrary to our expectations, the analysis of SCFA in the cecal contents revealed no significant differences between SHR-CTL and SHR-SJ (Table 2). The succinate in the cecal contents from all rats of the control group was below the lower limit of quantitation in SHR-SJ, although that of the SJ group was detected in the same measurements.

## 4. Discussion

The present study first confirmed that SJ intake significantly suppressed the BP increase in SHRs but did not significantly change the BP in WKYs. Then, this study demonstrated that the gut microbiota changed with SJ intake. However, the measurement of SCFAs in the cecal contents did not show significant alterations by consuming SJ in SHRs.

In this study, the gut microbiota exhibited significant changes when SHRs were fed an SJ diet, as observed in the α-diversity and in the β-diversity (Figure 2 and Figure 3). Previous studies have observed changes in the composition of the gut microbiota resulting from the intake of dietary fiber contained in SJ [10,12,13,14], and of polysaccharides extracted from SJ [43,44]. However, to the best of our knowledge, there are very few studies observing the changes in the diversity or the composition of the gut microbiota by the intake of SJ itself [45,46]. In this study, we have found that feeding SJ itself to SHRs changed the species of gut microbiota and significantly increased the relative abundance of *Bacteroides acidifaciens* in the cecum (Figure 4c). There are two possible pathways through which *Bacteroides acidifaciens* affects BP. First, since *Bacteroides acidifaciens,* an alginate and laminaran-degrading bacterium, has been reported to produce acetate and propionate [47], we supposed that *Bacteroides acidifaciens* in the cecum may increase the productions of acetate and propionate by consuming the alginate and laminaran contained in SJ, thereby suppressing the BP increase in SHRs. Although no significant differences were observed in the amount of acetate and propionate in the cecal contents in SHRs fed SJ compared with those fed CTL in the present study, it is not contrary to our hypothesis, because their amounts may depend not only on production but also on the consumption of acetate and propionate in the intestine. Second, it has been reported that forced oral administration of *Bacteroides acidifaciens* decreased dipeptidyl Peptidase-4 and thus increased serum glucagon-like peptide-1 (GLP-1) in mice [48]. It has also been reported that GLP-1 exhibits a BP-lowering effect by activating GLP-1 receptor [49,50]. Taken together, the increase in *Bacteroides acidifaciens* due to SJ intake may suppress the rise of BP in SHRs via increasing GLP-1. Since no studies have reported the effects of *Bacteroides acidifaciens* supplementation on hypertension as far as we know, further studies on it are necessary.

This study showed that *Akkermansia muciniphila* was further decreased with the ingestion of SJ (Figure 4c). However, previous studies have reported that the relative abundance of *Akkermansia* was decreased in SHRs compared with WKYs [29]. In addition, the administration of losartan to SHR has been reported to result in lowering BP compared with controls, along with an increase in the relative abundance of the *Akkermansia* genus [51]. On the other hand, *Akkermansia muciniphila* and *Bacteroides acidifaciens* possess the property of degrading mucin in the intestinal tract, releasing carbon and nitrogen, and promoting the growth of goblet cells and other bacteria [52]. Thus, the competition among *Akkermansia muciniphila*, *Bacteroides acidifaciens*, and other species might suppress the proliferation of *Akkermansia muciniphila*. 

The administration of *Faecalibacterium prausnitzii*, known as a probiotic, has been reported to attenuate atherosclerosis in Apo E^−/−^ mice [53]. This may be associated with an increase in intestinal barrier function due to the upregulation of zonula occludens-1 and mucin-2, as well as a decrease in inflammatory cytokines in the serum. However, in the present study, because no difference in the abundance of *Faecalibacterium prausnitzii* was observed in the cecal contents between CTL and SJ groups, we could not elucidate the role of *Faecalibacterium prausnitzii* in supressing BP elevation induced by SJ intake in SHRs.

The SJ intake did not significantly increase the concentrations of acetate, butyrate, and propionate in the cecal contents (Table 2). As described above, this may be the result of increased consumption along with production of these SCFAs. On the other hand, it has been reported that the expression of the butyrate transporter Slc5a8 in the colon and the expression of SCFAs receptors GPR 41 and 43 was decreased in the brain hypothalamus in SHRs [21]. As well, it has been reported that the intake of foods with antihypertensive effects increases the expression of GPR 41 while decreasing the expression of Olfr 78 [54]. Moreover, despite the higher concentration of butyrate in the cecal contents of SHRs compared with WKYs, the concentration of butyrate in serum has been reported to be low, suggesting the inhibition of butyrate absorption in the colon in SHRs. Thus, SJ intake might suppress BP elevation through changes in the expression of SCFA transporters and/or receptors. 

The present study showed that succinate was detected in the cecal contents of the control group but was below the lower limit of quantitation in the SJ group in SHRs (Table 2). Succinate is a ligand for GPR 91, the activation of which in the kidney mediates the release of renin [55]. Intravenous administration of succinate to Sprague Dawley (SD) rats has been reported to increase renin activity and to elevate MAP, which effect did not occur in SD rats that underwent bilateral nephrectomy [56]. The increase in BP caused by succinate has been reported to be abolished in GPR 91 knockout mice, suggesting that renal GPR 91 is involved in the increase in BP caused by succinate [56]. It has been also reported that SHRs have a higher plasma succinate concentration than the control WKYs [57]. Thus, if the decrease in the amount of succinate in the cecum leads to a decrease in the activity of GPR 91 in the kidney, the BP increase in SHRs may be suppressed by decreasing the renin-angiotensin system (RAS). Therefore, the inhibition of succinate by SJ intake might be involved in the mechanism of decreasing BP in SHRs fed SJ, although it may not be a major pathway.

One of the other possible mechanisms by which SJ intake suppresses BP elevation via gut microbiota is that SJ intake reduces the intestinal putrefaction products, including indoxyl sulfate and p-cresyl sulfate in serum, which may increase BP via the activation of the RAS. It has been reported that rats fed a diet containing 2% (*w*/*w*) laminaran or alginate demonstrated a decrease in the concentration of intestinal putrefaction products such as indole, phenol, and ammonia in feces and intestinal contents [12,14]. Intracolonic administration of indole and intravenous administration of its metabolite indoxyl sulfate were reported to increase BP in normotensive rats [58]. It has been reported that indoxyl sulfate, as well as p-cresol and p-cresyl sulfate, derived from the gut microbiota is associated with the activation of the renal RAS [59]. As well, a decrease in serum p-indoxyl sulfate and p-cresyl sulfate has been observed with the intake of inulin, a type of dietary fiber [60]. Therefore, it is possible that the reduction in indoxyl sulfate and p-cresyl sulfate in serum may be related to the mechanism by which SJ intake suppresses BP elevation. However, in this study, we have not observed intestinal putrefaction products and the fermentation of SJ, which should be investigated in the next step.

In addition, previous studies reported that the ACE inhibitory activity of SJ intake was observed in vitro [8]. It was reported that the plasma angiotensin. II concentration decreased compared to the vehicle group when alginate, one of the dietary fibers contained in SJ, was continuously administered to SHRs [10]. Although it is possible that ACE inhibition occurs in SJ intake in SHRs as well, ACE inhibitory activity in vitro is not always directly related to an antihypertensive effect. Thus, as investigated in other previous studies [4], demonstration of the antihypertensive effect of SJ intake in vivo will be necessary in further studies.

Alginate, one of the main components of SJ, has been observed to suppress BP elevation in SHRs or 2K1C rats, respectively [10,11,60]. It has been reported that promoting sodium excretion is a potential mechanism for the effect of potassium alginate ingestion in suppressing the increase in BP. However, our previous study suggested that the promotion of fecal sodium excretion by SJ intake may not be one of the major mechanisms for attenuating hypertension [7]. This was because no significant correlation was observed between the intake amount of alginate contained in various seaweeds and the suppression of BP elevation. Additionally, fecal sodium excretion was found to be very small in comparison to urine excretion [7]. Recently, we proposed one of the mechanisms behind this, which is the restoration of intestinal barrier function in 2K1C rats, by having observed an alleviation in the decreases in gut tight junction protein expression and the number of goblet cells in 2K1C rats fed alginate [11]. Therefore, the intake of SJ containing alginate may attenuate a decrease in intestinal barrier function in SHRs. Furthermore, in a recent study, high-fat-induced non-alcoholic fatty liver disease model rats were administered 2.5 g/kg BW of SJ daily for 8 weeks, resulting in a decrease in the malondialdehyde level in the liver and an increase in glutathione peroxidase and superoxide dismutase activities [61], suggesting the antioxidant effects of SJ intake may contribute to the suppression of BP elevation.

In summary, the present study demonstrated that SJ intake suppressed BP elevation and changed gut microbiota in SHRs. It especially increased *Bacteroides acidifaciens*, which might be involved in suppressing the BP elevation in SHRs.

## 5. Conclusions

SJ intake suppressed the increase in BP and altered the gut microbiota composition.

## Figures and Tables

**Table 1:** Effects of SJ on gut microbiota composition at the phylum level in SHRs.

Phylum	Relative Abundance (%)		*p* Value
	CTL	SJ	
*Firmicutes*	65.0 (58.2–70.6)	60.9 (60.2–66.2)	0.548
*Bacteroidetes*	30.1 (23.2–37.0)	34.5 (30.8–36.9)	0.548
*Verrucomicrobia*	1.8 (1.3–2.8)	0.6 (0.3–1.2)	0.016 *
*Protepbacteria*	1.4 (1.2–2.4)	2.0 (1.7–2.4)	0.222
*Actinobacteria*	0.9 (0.6–2.6)	0.2 (0.1–0.5)	0.032 *

Data shows median (75th and 25th percentiles). * *p* < 0.05 by Mann–Whitney U test. The following See legend of Figure 1 for abbreviations.

**Table 2:** SCFAs concentration in SHRs fed CTL or SJ.

SCFAs	Mean Concentration (mg/g)		*p* Value
	CTL	SJ	
Succinate	0.05 ± 0.01	N/D	-
Lactate	0.22 ± 0.07	0.06 ± 0.02	0.059
Acetate	2.76 ± 0.17	2.55 ± 0.13	0.356
Propionate	0.73 ± 0.09	0.69 ± 0.04	0.617
Butyrate	2.21 ± 0.30	2.43 ± 0.23	0.587

No significance by one-way ANOVA. See legend of Figure 1 for abbreviations.

## Data Availability

Data are contained within the article.
